# Revisiting Spirituality in Physical Therapy Practice: Perceptions of US Practitioners

**DOI:** 10.1007/s10943-025-02502-4

**Published:** 2025-12-29

**Authors:** Gale Lavinder, Penny Liberatos, Marc Campo, Erin Leegan, Meghan Moritz, Ryan Campo, Silvia Terziyski

**Affiliations:** 1 Department of Physical Therapy, New York Medical College, School of Health Sciences and Practice, Valhalla, NY USA; 2 Department of Public Health, New York Medical College, School of Health Sciences and Practice, Valhalla, NY USA; 3 Physical Therapy Program, Mercy University, Dobbs Ferry, NY USA; 4 Regain Physical Therapy, Pittsford, NY USA; 5 MedStar Health PT, Good Samaritan Hospital, Baltimore, MD USA; 6 UHS Binghamton General Hospital, Binghamton, NY USA; 7 CareOne, Wilmington, MA 01887 USA

**Keywords:** Spirituality, Education, Physical therapy, Patient-centered care

## Abstract

Little discussion of including the spiritual domain in physical therapy (PT) education in the US has occurred despite support from PT practitioners and students. The purpose of this study was to assess the perceptions of practicing physical therapists regarding the role that their own spirituality, prior professional education, and the usefulness of including spirituality in PT practice may play in several aspects of clinical practice. A random sample of 800 physical therapists working in acute/sub-acute rehabilitation settings selected from the APTA membership was surveyed. This cross sectional study achieved a response rate of 57.5% (n = 460). Outcomes consisted of 57 Likert-scale items focused on the role that spirituality might play in PT clinical practice. Internal consistency reliability of these items was excellent (α = .97). The importance of including spirituality in patient care (total score) was significantly related to respondents’ self-identified belief system (where religious/spiritual were more positive relative to atheist/agnostic/other) (*p* < 001/h2 = .178) andperceptions of usefulness of including spirituality in patient care (where positive perceptions related to positive outcomes) (*p* < .001/d = −1.856). Although those with prior exposure to spirituality through professional education tended to agree more with the outcomes, only three of the five topic areas were statistically significant, but the total score was not significantly related (*p *= .237/d = −.154). Study results contribute to the existing literature supporting the importance of addressing spirituality in PT education. Educating physical therapists in this area would enhance the profession’s goal of providing holistic patient-centered care and improving cultural competence.

**Supplementary Information:**

The online version contains supplementary material available at 10.1007/s10943-025-02502-4.

## Introduction

The role that spirituality can play in healthcare and how it may affect the rehabilitation process has been of particular interest to researchers over the last 20 years (Jones et al., 2018; Koenig et al., 2004a, 2004b; Wachholtz et al., 2007; Waldron-Perrine et al., 2011). A review of the literature offers no universal definition of spirituality (Abu-Raiya et al., 2015; Stephenson & Berry, 2015; Tanyi, 2002). However, the concept often embraces a search for meaning, connection to self and others, nature, a purpose in life, and an overall sense of wellness, which transcends individual and social circumstances offering support in times of personal strife and/or illness (Delgado, 2005; Victor & Treschuk, 2020). Religion, on the other hand, has been described as an extrinsic organized set of values, beliefs and practices in relationship with the transcendent and grounded in institutional standards (Sulmasy, 2009; Tanyi, 2006). Several authors have cited overlap between spirituality and religion (Baldacchino, 2015; Boswell et al., 2013; Hollins, 2005). Considered inclusive of religion and spirituality, the spiritual domain has been defined as intrinsic and/or extrinsic beliefs about spirituality, religion, faith, and/or moral development (Shepard & Jensen, 2002). Rather than delimit spirituality and religion, like Sargeant and Newsham (2012) we will refer to both spirituality and the spiritual domain as broad inclusion.

Incorporating spirituality in healthcare involves “serving the whole person—the physical, emotional, social and spiritual” (de Oliveira et al., 2019; Puchalski et al., 2014a, 2014b; Sargeant, 2009; Savel & Munro, 2014). This is consistent with the emphasis on patient-centered care, one of six domains of healthcare quality (Agency for Healthcare Research & Quality, 2018). Patient-centered care is intended to be responsive to patients’ needs and values with these preferences guiding health care decisions (Tzelepis et al., 2015). The patient-centered approach promotes biopsychosocial care including the spiritual domain, and views healing through understanding the whole person thereby enhancing the therapeutic relationship by offering compassion, empathy, healing and hope (Mathisen et al., 2015; Puchalski, 2013; Savel & Munro, 2014). This model, referred to as the biopsychosocialspiritual model by Sulmasy (2002), has been utilized in palliative care and more recently in pain medicine (Justice et al., 2023; Siddall et al., 2015).

The importance of the spiritual domain among Americans is supported by data from a nationally representative survey in 2014 indicating that 77% of US adults consider religion at least somewhat important in their lives (Pew Research Center, 2019). Furthermore, results from several studies have shown that: hospitalized patients with severe illness (29%) or near-death circumstances (50%) felt that physician spiritual interaction was important (MacLean et al., 2003); that they used faith to cope with illness (87%; Ellis et al., 2013); that addressing patient spirituality during hospitalization led to greater satisfaction with their physician (Uhelski et al., 2022); and that patients with advanced disease or serious medical conditions found it beneficial to talk about their spiritual beliefs/doubts (Fitch & Bartlett, 2019). Thus, patients seem to be positively predisposed to providers discussing spiritual concerns and feel that it would strengthen their trust in the provider (Puchalski, 2001).

From the provider’s standpoint, understanding patients’ spirituality may help them understand how patients perceive their condition, cope with illness, and make treatment decisions—all important components to patient-centered care (Puchalski, 2013). This understanding may benefit the quality of care, patient-provider relationship and improve medical adherence (Saguil & Phelps, 2012).

The significant role of spirituality in healthcare has also been recognized by several organizations: Commission on Accreditation of Rehabilitation Facilities (CARF, 2012), Joint Commission on Accreditation for Healthcare Organizations (JCAHO, 2019), World Health Organization (2012), American Physical Therapy Association (APTA, 2008), and American Occupational Therapy Association (2017). In fact, JCAHO requires a spiritual assessment to be conducted for all patients admitted to hospitals (The Joint Commission, 2010).

Specifically in physical therapy, the values of compassion/caring and professional duty support a clinician’s ability to understand the patient’s perspective, which would include the spiritual domain (APTA, 2007; Sargeant, 2009). The APTA considers spirituality as part of cultural competence and person-centered care which are a part of best practice to address an individual’s culture, needs and values (APTA, 2008). The APTA has also adopted the International Classification of Functioning, Disease and Health (ICF) model. This model has become more important since it includes contextual factors – personal and environmental—(including the spiritual domain) as part of understanding an individual’s health and well-being because they influence function and disability (APTA, 2008; Offenbacher et al., 2011; Wade & Halligan, 2017). Similarly, a recent study has identified the spiritual domain as a social determinant of health, thus emphasizing contextual factors in patient-centered care (Garcia et al., 2024) and aligning with APTA’s vision for educating physical therapists in the twenty-first century (Jensen et al., 2019).

This professional expectation is extended through the accreditation process where such curricular standards are part of the review process (Commission on Accreditation of Physical Therapy Education [CAPTE], 2024). For example, CAPTE Evaluative Criteria 7D5: *Practice in a manner consistent with APTA Core Values*; 7D8: *Identify, respect and act with considerations for patients’/clients’ differences, values, preferences, and expressed needs in all professional activities*. Criteria state that culture encompasses one’s language, actions, beliefs, values as well as racial, ethnic, religious or social groups.

The purpose of this study is to assess the relationship of three factors on the perceptions and attitudes of physical therapists regarding the inclusion of spirituality in physical therapy (PT) practice. Specifically, three aims are proposed for this study regarding the influence of: 1) the practitioner’s own belief system; 2) prior exposure to spirituality through professional education; and 3) the perceived usefulness of incorporating spirituality in patient care on their level of agreement with the role of spirituality in several areas of PT clinical practice. Based on the results of this and prior studies, the appropriateness of recommending a more explicit inclusion of the spiritual domain in PT education is considered.

## Literature Review

Study findings across a variety of health disciplines have demonstrated that spiritual wellness is positively related to quality of life or recovery from illness/injury/surgery (Abrams et al., 2018; Carey & Mathisen, 2018; Kotlińska-Lemieszek et al., 2022; Naghi et al., 2012; Smothers & Koenig, 2018; Waldron-Perrine et al., 2011). Spirituality was related to decreased lengths of stay, fewer post-op complications, fewer medical complaints, and lower mortality rates in patients recovering from cardiac conditions (Mouch & Sonnega, 2012; Naghi et al., 2012; von Flach et al., 2023). Studies have also correlated the inclusion of the spiritual domain in clinical care with improvements in depression, stress/anxiety, chronic pain, PTSD, and post-stroke aphasia (Ferreira-Valente et al., 2022; Glover-Graf et al., 2007; Laures-Gore et al., 2018; Smothers & Koenig, 2018); it is also seen as a protective factor among adults surviving burn injuries (Abrams et al., 2018; Askay and Magyar-Russell, 2009).

It is important to consider how practicing health professionals perceive this importance. Prior studies have shown that not only is awareness/recognition of spirituality important in the clinical context, but spirituality is an important component of health and well-being, improved health outcomes, allows clinicians to provide patient-centered care, addresses patient concerns, and is an important component in patient adjustment to life-changing events (Berg et al., 2013; Egan & Swedersky, 2003; Jones et al., 2018; Oakley et al., 2010; Sargeant, 2003). Few participants in these studies felt addressing spirituality with patients was either not in their scope of practice or could affect the patient negatively.

Studies exploring the perspectives of spirituality among PT and occupational therapy (OT) students support the importance of addressing spirituality in clinical practice to motivate the patient and influence positive outcomes (Highfield & Osterhues, 2003), enhance the patient-therapist relationship/coping (Sargeant & Newsham, 2012), and foster holistic care (Gang et al., 2022; Mthembu et al., 2016). In addition, students felt that they gained an appreciation for different spiritual/religious views and that spiritual issues should be explored in professional education (Gang et al., 2022; Mthembu et al., 2015; Tapley et al., 2012) due to a need for role models and experience in how to address the spiritual domain within the clinical context. These themes also emerged among OT students as suggestions for development of curricula on this topic (Thompson & MacNeil, 2006).

Despite the acknowledgement and support of professional organizations to address the spiritual domain in patient care, physical therapists and other health care providers have highlighted some challenges and barriers to implementation. These barriers include challenges in finding time, lack of provider comfort, and lack of training to incorporate spiritual concerns into patient-centered care (Collins et al., 2009; D’Souza, 2007; McEvoy et al., 2013; Oakley et al., 2010; Saguil & Phelps, 2012; Vermandere et al., 2011; Wu et al., 2012). In addition, some clinicians feel that it is outside their scope of practice since they have not received academic training in this area and therefore feel that they are not prepared to address these concerns clinically (Anandarajah & Hight, 2001; Ellis et al., 2002; Franzen, 2018; Morris et al., 2012; Oakley et al., 2010; Saguil & Phelps, 2012; Taylor et al., 2000). These barriers likely influence practitioners’ degree of confidence in their ability to have such discussions with patients.

Among practicing physical therapists, very few studies have assessed practitioners’ views on the role that spirituality might play in PT practice. Studies that do exist have either had small sample sizes and/or used broad statements regarding therapists’ beliefs and barriers to incorporating spirituality in their practice (Oakley et al., 2010; Sargeant, 2003). Developing a thorough understanding regarding the role that the spiritual domain might offer during interactions with patients, can affect PT practitioners and patient health outcomes.

## Methods

### Study Design and Participants

This was a cross-sectional study of practicing physical therapists who were members of the American Physical Therapy Association (APTA). Among the APTA membership list, physical therapists who worked in acute or sub-acute rehabilitation facilities were selected, then a random sample of 800 was drawn (through random number generation) from that list. Acute/sub-acute rehabilitation settings were selected since services are provided daily over a period of several weeks, allowing therapists to develop a deeper connection with their patients. Also, many patients in these settings are receiving PT services after a potentially life-changing diagnosis (e.g., CVA, SCI, amputation) which may allow the topic of spirituality to present itself. It was felt that the typically less frequent interactions of outpatient settings would be less likely to generate these close connections.

### Survey Development

Since there were only two prior studies focused on spirituality with PT practitioners—one qualitative (Sargeant, 2003) and the other using a modified instrument originally designed for physicians (Oakley et al., 2010)—it was felt that a more comprehensive measure focused on PT practice would be important to develop. A survey was developed by two of the authors (GL&MC). The content was developed through a review of the literature related to spirituality in health care, existing measures of spiritual well-being and beliefs, and in consultation with Sargeant (Ellis et al., 1999; Hatch et al., 1998; McCauley et al., 2005). The focus of this survey was practicing in-patient physical therapists and how they perceive the role of spirituality in clinical practice. This process yielded five topic areas for the survey questions.

The five identified topic areas were: Topic 1: Description of Spirituality (participants’ views on spirituality); Topic 2: Spirituality and PT Practice (spirituality and PT practice as a service profession); Topic 3: Spirituality and the Patient (effects of spirituality on patients); Topic 4: Spirituality and the Physical Therapist (influence of spirituality on a therapist’s development); and Topic 5: Spirituality and Outcomes (influence of spirituality on rehabilitation outcomes). These topics were guided by key issues identified in the literature as related to spirituality in clinical practice. The five topic areas were also confirmed by clinical judgment as described below.

Two sources of expertise were utilized to establish face and content validity. The first expert was Dr. Darina Sargeant who had conducted the qualitative study of physical therapists (2003) and the survey of PT students (Sargeant & Newsham, 2012) and who is considered a leading researcher/author in the field of spirituality and PT practice. The second expert source consisted of eight physical therapists who served as clinical faculty members of the PT Clinical Education Advisory Board at St. Louis University. They each had 5–25 years of experience as physical therapists in a variety of settings. Face validity was assured through responses to questions from the two expert sources as to whether the content of the survey appeared to measure spirituality in PT practice. Content validity was established through responses to questions of the two expert sources as to the appropriateness of the topics and the breadth of items covered under those topics. The survey was pilot tested by two physical therapists at a rehabilitation center and modified accordingly (e.g., clarification of wording and response categories). Approximate time to complete the survey was 15 min as determined through pilot testing.

The total survey consists of 67 items, with 64 closed-ended and 3 open-ended questions. Questions about spirituality (*n* = 57) focused on the five identified topics described above: These statements used a five-point Likert scale (Strongly agree/ Agree/ Undecided/ Disagree/Strongly disagree), where 1 = strongly agree and 5 = strongly disagree. The internal consistency reliability for all spirituality items (*N* = 57) was found to be excellent with a Cronbach’s alpha of .97. Reliability for the five topic scores was also good to excellent: Spirituality Description (α = .77); Spirituality and PT Practice (α = .80); Spirituality and Patient (α = .96); Spirituality and Therapist (α = .83); and Spirituality and Outcomes (α = .94).

Respondents’ self-identified belief system was assessed by: “I consider myself… (Religious/Spiritual/Agnostic/Atheist/ Humanist/Other; multiple responses possible). Spirituality was defined as “an internal experience that provides a sense of meaning and purpose in life and connectedness with self and other” and religion was defined as “an extrinsic organized set of standards and beliefs grounded in institutional standards”. The remaining response categories were not defined by the survey. This approach was also used by Sargeant and Newsham (2012).

Since multiple responses were possible for self-identified belief system, participants were classified based on the following priority order: Religious when selecting that response (regardless of other categories selected); Spiritual when selecting that response (regardless of other categories selected but not religious); Humanist (regardless of other categories selected but not religious or spiritual); Other if they identified as agnostic/atheist/other (but not religious, spiritual, humanist).

This decision tree was based on the following: 1) religious is viewed as containing a spiritual element that is expressed both internally and externally (Koenig, 2002), so that was seen as broader and given priority over spiritual; 2) spiritual is seen as a connection to a higher power; 3) although humanist does not believe in a supernatural power, there is a belief in human nature, reason, individualism and a compassion for human-kind; 4) finally, atheist-agnostic and other do not have the above elements, so that was created as the last category.

Respondents were also asked about prior exposure to spirituality through professional education (Yes/No), whether considering patients’ spirituality in practice is useful (Yes/No), and demographic characteristics which consisted of age, gender, years as a physical therapist and self-identified belief system (discussed above). Three open-ended questions inquired about the meaning of spirituality to respondents, asked respondents to expand on why they thought that considering spirituality in PT practice might or might not be useful, and allowed for any additional comments (see Supplemental Material).

### Procedures

A cover letter describing the study, coded survey and self-addressed stamped/coded envelope were mailed to all selected participants. Three follow-up postcard reminders were sent to non-respondents. Respondents were entered into a random drawing for twenty $20 gift certificates.

### Data Analysis

Spirituality items within each of the five topics (i.e., spirituality description (Topic 1)–*n* = 5; spirituality and PT practice (Topic 2)–*n* = 14; spirituality and patient (Topic 3)–*n* = 20; spirituality and therapist (Topic 4)–*n* = 7; and spirituality and patient outcomes (Topic 5)–*n* = 11) were summed to create topic scores. All 57 items were also summed to create a total score. The total and five topic scores constituted the dependent variables for the study.

Three independent variables were used in this study. The first was the respondent’s self-identification of their belief system [Aim 1]. Four categories were used in analysis: Religious/Spiritual/Humanist/Other (consisting of agnostic + atheist + other).

The second independent variable was whether respondents had prior exposure to spirituality through PT or continuing education (Yes/No, with any exposure coded as Yes) [Aim 2]. No distinction was made between having some exposure through PT education or through continuing education because no details regarding type of exposure (e.g., course, lecture, webinar), duration, or frequency of education were obtained. Thus, this variable represents any exposure in a professional educational context. The third independent variable was whether participants thought that considering the spirituality of the patient in PT practice was useful (Yes/No) [Aim 3].

Descriptive analyses present frequencies, percents, means and standard errors of variables. Comparisons of mean topic scores across the three independent variables and demographic characteristics were made using independent sample t-test (for prior exposure, usefulness, age, and years working as a physical therapist) and one-way analysis of variance (or Brown-Forsythe test) with Hochberg’s GT2 post hoc tests (for self-identified belief system, age and years as a PT). Effect size was assessed by Cohen’s *d* (for *t*-test) and eta^2^ (for one-way analysis of variance). Since six dependent variables were included in this analysis, a Bonferroni correction was made to adjust for Type I error. Thus, for a *p* value of .05, the adjusted *p* value that was used in this study is .008 (.05/6 = .008), resulting in the criterion of *α* < .008 for achieving statistical significance. These analyses were performed using SPSS, v29.

Content analysis was performed for the three open-ended questions. The primary investigator developed an initial set of categories based on the literature in spirituality and categories that emerged directly from review of a 10% random sample (*n* = 47) of all surveys (e.g., patient choice to discuss spirituality—initiated by patient or by therapist). These initial categories formed the coding structure for reviewing the remaining surveys. Categories were added/modified as necessary (e.g., barriers to including spirituality, comfort level to discuss, used as coping mechanism) as more surveys were reviewed, discrepancies were resolved as part of a “dialogue and consensus process” (Cofie et al., 2022). Ultimately, 21 categories were finalized and assigned numerical codes (see Table 5 for categories). Each category was specifically defined to standardize classification among researchers.

Groups of five to seven randomly selected surveys were coded by the primary investigator and four student researchers to develop consistency in assigning codes. Discrepancies in coding were resolved through the “dialogue and consensus process” (Cofie et al., 2022). Inter-coder consistency was determined after multiple trials of coding by all five researchers. Each trial was averaged until at least 80% agreement was reached (O’Connor & Joffe, 2020). Once this was achieved, all remaining surveys were divided among the four student researchers to code individually. The primary investigator performed a random check of student coding to assure accuracy. Verbatim responses that were subjectively deemed interesting or compelling by the researchers were recorded separately.

### Ethical Considerations

This study was performed in line with the principles of the Declaration of Helsinki. Approval was granted by the Institutional Review Board of Mercy College. To preserve the anonymity of survey respondents, the returned survey was considered consent to participate.

## Results

### Respondent Demographics

A total of 460 physical therapists responded to the survey, for an overall 57.5% response rate (460/800). Table 1 shows the demographic characteristics of these respondents. Almost 80% were female and 55.7% were aged 35–44. Ninety percent of participants had more than 10 years of PT work experience, with 48.1% having 11–15 years’ experience. The majority (61.5%) considered themselves religious and an additional 29.8% described themselves as spiritual. The remainder self-described as humanist (3.3%) or other (5.5%). Most (76.9%) reported not having had prior education regarding spirituality.

**Table 1:** Demographic characteristics of study sample (N = 460)

Variable	N	%
*Sex*		
Male	92	20.1
Female	365	79.9
*Age (years)*		
20–34	39	8.4
35–44	256	55.7
45–54	121	26.3
55 +	44	8.9
*Years worked as PT*		
0–10	51	11.2
11–15	220	48.1
16–20	73	16.0
> 20	113	24.7
*Self-identified belief system*		
Religious	281	61.5
Spiritual	136	29.8
Humanist	15	3.3
Other^a^	25	5.5
*Prior exposure to spirituality education*		
Yes	106	23.1
No	352	76.9
*Useful to consider spirituality*		
Yes	408	93.2
No	30	6.8

^a^Other = Atheist, agnostic and other

When assessing the four categories reflecting the respondents’ self-identified belief system relative to demographic characteristics, there were no significant differences in the respondents’ belief system by sex, age or years worked as a PT. As shown in Table 2, a larger proportion of females considered themselves spiritual compared to males (31.6% vs. 22.8%), while a larger proportion of males identified as humanist or other (14.1% vs. 7.1%). The age groups that had the highest proportions reporting themselves as religious were those 35–44 (67.1%) followed by 20–34 (59.0%).

**Table 2:** Self-Identified belief system by demographic characteristics (N = 460)

Variable	Total	Religious		Spiritual		Humanist		Other^a^		*p*-value^b^
	N	N	%	N	%	N	%	N	%	
*Sex*	456	281	61.6	136	29.8	15	3.3	24	5.3	.032
Male	92	58	63.0	21	22.8	7	7.6	6	6.5	
Female	364	223	61.3	115	31.6	8	2.2	18	4.9	
*Age (years)*	457	281	61.5	136	29.8	15	3.3	25	5.5	.008
20–34	39	23	59.0	12	30.8	0	0.0	4	10.3	
35–44	255	171	67.1	67	26.3	7	2.7	10	3.9	
45–54	121	64	52.9	44	36.4	3	2.5	10	8.3	
55 +	42	23	54.8	13	31.0	5	11.9	1	2.4	
*Years Worked as PT*	456	280	61.4	136	29.8	15	3.3	25	5.5	.105
0–10	51	31	60.8	12	23.5	2	3.9	6	11.8	
11–15	220	146	66.4	59	26.8	7	3.2	8	3.6	
16–20	72	36	50.0	31	43.1	1	1.4	4	5.6	
> 20	113	67	59.3	34	30.1	5	4.4	7	6.2	

^a^Consists of Agnostic, Atheist, and Other

^b^Comparisons made by chi-square statistic; adjusted α < .008

The respondent’s self-identified belief system was also assessed with the other independent variables (results not shown). Examining the usefulness of spirituality in patient care with the respondent’s self-identified belief system shows that a higher proportion of those identifying as religious/spiritual (95.2%, 95.3%, respectively) compared to humanist/other (78.6%, 68.2%, respectively) thought that spirituality in PT care would be useful (*p* < .001). However, there was no relationship between belief system categories and prior education (religious-21.8%; spiritual-27.9%; humanist-26.7%; other-12.0%; *p* = .274). Similarly, most respondents thought that it was useful to include spirituality in patient care regardless of whether they had prior education in spirituality (92.2%) or not (93.4%; *p* = .673;).

Mean (total and 5 topic) scores were compared by sex, age and years worked as a PT. There were no significant differences for any of these variables. Effect sizes were low for sex (Cohen’s *d* ranged from .02 to .14), age (ƞ^*2*^ ranged from .001 to .01) and years worked (ƞ^*2*^ ranged from .004 to .03; results not shown).

#### Aim 1

Table 3 presents the mean outcome scores by the respondent’s self-identified belief system. The degree of agreement with statements about the role of spirituality in practice (total score) was significantly related to respondents’ self-identified belief system. Thus, those considering themselves to be religious (Mean = 104.9/ *SE* = 1.8) had lower total means (i.e., agreed more with the items) than those considering themselves as spiritual (Mean = 113.6/*SE* = 2.8), or humanist (Mean = 145.2/*SE* = 8.4), or other (Mean = 159.1/*SE* = 10.4; *p* < .001*/*η^*2*^ = .18).

**Table 3:** Comparison of mean outcome topic scores by self-identified belief system*

Variable	Spirituality description						Variable	Spirituality in PT Practice					
	N	Mean (SE)	F	df	*p*-value	Eta^2^		N	Mean (SE)	F	df	*p*-value	Eta^2^
Belief system	453		15.67	3,449	< .001	.10	Spirituality	437		19.68	3,433	. < .001	.12
Religious^d^	279	8.0 (.2)					Religious^c,d^	269	26.2(.4)				
Spiritual^d^	134	8.4 (.2)					Spiritual^c,d^	130	26.6(.6)				
Humanist	15	10.1 (.8)					Humanist^a,b^	15	33.1(1.4)				
Other^a,b^	25	11.4 (.5)					Other^a,b^	23	36.4(1.9)				

Variable	Spirituality + patient						Variable	Spirituality + physical therapist					
	N	Mean (SE)	F	df	*p*-value	Eta^2^		N	Mean (SE)	F	df	*p*-value	Eta^2^
Belief system	437		16.77	3,57.96	< .001	.13	Spirituality	442		19.27	3,438	< .001	.12
Religious^c,d^	271	37.8(.6)					Religious^c,d^	272	14.8(.2)				
Spiritual^c,d^	131	40.5(.9)					Spiritual^d^	132	15.5(.4)				
Humanist^a,b^	13	50.7(2.6)					Humanist^a^	13	18.9(.9)				
Other^a,b^	22	54.6(3.4)					Other^a,b^	25	20.2(.9)				

Variable	Spirituality + outcomes						Variable	Total spirituality score					
	N	Mean (SE)	F	df	*p*-value	Eta^2^		N	Mean (SE)	F	df	*p*-value	Eta^2^
Belief system	354		15.94	3,350	< .001	.12	Spirituality	318		22.73	3,314	< .001	.18
Religious^c,d^	220	21.2(.4)					Religious^c,d^	200	104.9(1.8)				
Spiritual^d^	107	23.0(.6)					Spiritual^d^	96	113.6(2.8)				
Humanist^a^	12	28.8(2.1)					Humanist^a^	9	145.2(8.4)				
Other^a,b^	15	31.7(2.5)					Other^a,b^	13	159.1(10.4)				

*Comparisons made by one-way ANOVA, except for “Spirituality + Patient”, where Brown-Forsythe statistic was used due to unequal variances; adjusted α < .008

Post hoc analyses performed using Hochberg GT2. Significant comparisons shown below.

^a^Significantly different from Religious

^b^Significantly different from Spiritual

^c^Significantly different from Humanist

^d^Significantly different from Other (includes atheist, agnostic, other)

Post hoc tests showed that the categories of religious and spiritual were not significantly different from each other (*p* = .054). Similarly, humanist and other did not differ from each other (*p* = .794). However, religious and spiritual were each significantly different from humanist (*p* < .001, *p* = .005, respectively) and other (*p* < .001, *p* < .001, respectively).

This same significant pattern was also manifest in each of the five topic mean scores, where those identifying as religious had the lowest mean, followed in order by those in the spiritual, humanist, and other categories (Topic 1—*p* < .001/η^*2*^ = .10; Topic 2—*p* < .001/ η^*2*^ = .12; Topic 3—*p* < .001/ η^*2*^ = .13; Topic 4—*p* < .001/ η^*2*^ = .12; Topic 5—*p* < .001/ η^*2*^ = .12). These results were all statistically significant and the magnitude of the effects are considered medium for the individual topics (explaining 10–13% of the variance) and large for the total score (explaining 18% of the variance), thereby indicating a stronger relationship. This interpretation is based on Cohen’s (1988) benchmarks of effect size where medium effect is ƞ^2^ = .06 − .139 and ƞ^2^ ≥ .14 as a large effect size.

Post hoc tests for each of the five outcome topic areas for the most part showed a similar pattern, where religious and spiritual were not significantly different from each other (Topic 1—*p* = .444; Topic 2—*p* = .995; Topic 3—*p* = .119; Topic 4—*p* = .445; Topic 5—*p* = .130). Similarly, humanist and other were not significantly different from each other (Topic 1—*p* = .596; Topic 2—*p* = .609; Topic 3—*p* = .879; Topic 4—*p* = .881; Topic 5—*p* = .820). However, religious was significantly different from humanist (Topic 1—*p* = .011; Topic 2—*p* = .001; Topic 3—*p* < .001.; Topic 4—*p* = .001; Topic 5—*p* = .001) and other (Topic 1—*p* < .001; Topic 2—*p* < .001; Topic 3—*p* < .001; Topic 4—*p* < .001; Topic 5—*p* < .001) and spirituality was significantly different from humanist for two out of five topics (Topic 1—*p* = .097; Topic 2—*p* = .003/; Topic 3—*p* = .007/; Topic 4—*p* = .014; Topic 5—*p* = .030) and significantly different from other for all five topics (Topic 1—*p* < .001/; Topic 2—*p* < .001; Topic 3—*p* < .001; Topic 4—*p* < .001; Topic 5—*p* < .001).

#### Aim 2

Table 4 presents mean outcome topic scores for prior exposure to spirituality education. Respondents who had prior exposure to spirituality through professional education tended to agree more with the spirituality statements than those who had no exposure. However, these mean differences were statistically significant for only three of the five topics (Topic 1—Mean = 7.7/*SE* = .3 vs. Mean = 8.6/*SE* = .2, *p* = .006/ *d* = −.31; Topic 2—Mean = 25.3/*SE* = .8 vs. Mean = 27.6/*SE* = .4, *p* = .006/ *d* = −.32; Topic 3—Mean = 39.2/*SE* = 1.9 vs. Mean = 40.2/*SE* = .6, *p* = .447/ *d* = −.09; Topic 4—Mean = 14.4/*SE* = .4 vs. Mean = 15.7/*SE* = .2, *p* = .002/ *d* = −.35; Topic 5—Mean = 22.0/*SE* = .8 vs. Mean = 22.7/*SE* = .4, *p* = .418/ *d* = −.10; Total—Mean = 107.7/*SE* = 3.6 vs. Mean = 112.2/*SE* = 1.9, *p* = .237/ *d* = −.15).

**Table 4:** Comparison of mean outcome topic scores by prior exposure to spirituality education and perceived usefulness of incorporating spirituality into physical therapy practice^a^

Variable	Spirituality description						Spirituality in PT practice					
	N	Mean (SE)	t	95% CI	*p*-value	Cohen’s d	N	Mean (SE)	t	95% CI	*p*-value	Cohen’s d
Prior exposure to spirituality education			− 2.77	− 1.46, − .25	.006	− .31			− 2.78	− 3.97, − .68	.006	− .32
Yes	106	7.7(.3)					97	25.3(.8)				
No	348	8.6(.2)					341	27.6(.4)				
Useful to consider spirituality			− 3.34^b^	− 3.81, − .92	.002	− .86			− 8.68	− 14.12, − 8.91	< .001	− 1.73
Yes	404	8.2(.1)					382	26.1(.3)				
No	30	10.6(.7)					27	37.6(1.1)				

Variable	Spirituality + patient						Spirituality + physical therapist					
	N	Mean (SE)	t	95% CI	*p*-value	Cohen’s d	N	Mean (SE)	t	95% CI	*p*-value	Cohen’s d
Prior exposure to spirituality education			− .76	− 3.54, 1.56	.447	− .09			− 3.07	− 2.28, − .50	.002	− .35
Yes	102	39.2(1.9)					103	14.4(.4)				
No	336	40.2(.6)					339	15.7(.2)				
Useful to consider spirituality			− 7.99	− 20.28, − 12.27	< .001	− 1.54			− 6.53	− 6.15, − 3.30	< .001	− 1.24
Yes	389	38.4(.5)					392	14.9(.2)				
No	29	54.7(2.5)					30	19.7(.8)				

Variable	Spirituality + outcomes						Total spirituality score					
	N	Mean (SE)	t	95% CI	*p*-value	Cohen’s d	N	Mean (SE)	t	95%CI	*p*-value	Cohen’s d
Prior exposure to spirituality education			− .81	− 2.41, 1.00	.418	− .01			− 1.186	− 12.12, 3.01	.237	− .15
Yes	90	22.0(.8)					78	107.7(3.6)				
No	265	22.7(.4)					241	112.2(1.9)				
Useful to consider spirituality			− 7.10	− 13.08, − 7.40	< .001	− 1.56			− 8.02	− 61.15, − 37.05	< .001	− 1.86
Yes	316	21.7(.4)					283	106.9(1.6)				
No	22	32.0(1.6)					20	156.0(7.2)				

^a^Comparisons made by independent t-test; adjusted α < .008

^b^Unequal variances assumed; equal variances assumed for all other variables

#### Aim 3

Table 4 also presents mean outcome topic scores for the perceived usefulness for considering the patient’s spirituality in PT practice. Those reporting that considering spirituality in patient care would be useful were significantly more likely to agree with the spirituality outcome statements. For example, the total outcome mean score for those who thought it useful was significantly lower than those who did not think it useful (Mean = 106.9/*SE* = 1.6 vs. Mean = 156.0/*SE* = 7.2, *p* < .001/ *d* = −1.86). This was true for all topic means as well. Comparisons for each of the five outcome topics are: Topic 1—Mean = 8.2/*SE* = .1 versus Mean = 106./SE = .7, *p* = .002/ *d* = −.86; Topic 2—Mean = 26.1/*SE* = .3 versus Mean = 37.6/*SE* = 1.1, *p* < .001/ *d* = −1.73; Topic 3—Mean = 38.4/*SE* = .5 versus Mean = 54.7/*SE* = 2.5, *p* < .001/ *d* = −1.54; Topic 4—Mean = 14.9/*SE* = .2 versus Mean = 19.7/*SE* = .8, *p* < .001/ *d* = −1.24; Topic 5—Mean = 21.7/*SE* = .4 versus Mean = 32.0/*SE* = 1.6, *p* < .001/ *d* = −1.56.

### Qualitative Analysis

The results of the content analysis are shown in Table 5. Eighty percent of responses indicated that it was the patient’s choice as to whether to incorporate spirituality in their care. Ten percent of responses highlighted the importance of spirituality in motivating the patient. A very small minority of responses (1.3%) indicated that it was “dangerous” for a PT to approach the topic of spirituality. Regarding what spirituality means to themselves as therapists, responses were consistent with the definitions used in this study: “life purpose”, “higher power”, “God/Creator”.

**Table 5:** Qualitative findings regarding the role of spirituality

Qualitative category	N^a^(Responses)	%
*Usefulness to consider patient spiritual dimension*	555	
Patient choice	444	80.0
Motivation of patient	45	8.1
Negative impact/dangerous	7	1.3
Refer to ministry	7	1.3
Other	52	9.4
*Meaning of spirituality to therapist*	468	
Self-understanding/development	88	18.8
Life purpose	77	16.4
Higher power	65	13.9
God/Creator	63	13.5
Connection with others	56	12.0
Inner peace	27	5.8
Jesus Christ/Lord	21	4.5
Body/Mind/Spirit	18	3.8
Spirituality & Religion are different	10	2.1
Other	43	9.2
*Other: Comments*	136	
Patient choice	36	26.5
Beneficial	19	14.0
Barriers (therapist)	14	10.3
Refer to ministry	9	6.6
Not in PT scope of practice	7	5.1
Other	51	37.5

^a^Each of the three open-ended questions had a different number of responses as indicated in this column. Each individual could have provided multiple responses or no responses at all to these questions

Some responses focused on the importance of spirituality in their own lives. For example, “self-understanding/development” (18.8%), “connection with others” (12.0%), and “inner peace” (5.8%). The most common responses among “further comments” were that addressing spirituality depends on the patient (26.5%), that it is beneficial to PT practice, (14.0%), that therapists face barriers to incorporating spirituality (10.3%), and that it is not within PT scope of practice (5.1%). Specifically, barriers that respondents identified were lack of time, impact on productivity and feeling fearful or uncomfortable due to lack of training.

An examination of specific comments made by respondents found three main themes.

1. Spirituality as part of patient-centered holistic care.

Statements consistent with this theme are: “*I believe that spiritualism is part of the wholeness of a person, and is included in treatment*”; “*Spirituality is one aspect of treating the patient as a whole rather than parts*”; “*I believe strongly that this type of research is needed to help understand and treat the whole person*”; “*I feel that spirituality should be incorporated in patient-centered caregiving as it is a dimension that completes a person’s wholeness*”; and “*Considering the spiritual dimension of the patient helps me to encourage and motivate them*”.

(2) Spirituality in professional education.

This theme is exemplified by the following statements: “*I do not feel that it [spirituality] is my most important focus, b/c it is not the most of my training*”; “*Education on spirituality should definitely be included in PT education*”; and “*I haven’t seen any continuing education addressing spirituality, but would love to*”.

(3) Practical limitations to including spirituality.

Finally, the last theme is demonstrated by these statements—“*I am aware of it at work. There are limitations when the case load is high at times*” and “*It is a sensitive but important issue*”.

## Discussion

### Aim 1—Practitioner’s Own Belief System

Ninety percent of study participants self-identified as either religious or spiritual. This indicates an overwhelming majority of practitioner respondents see some role for religiosity/spirituality in their own lives. Similarly, findings from several studies across disciplines have shown that an individual’s own spirituality may give practitioners a greater understanding that in turn may enhance the therapeutic relationship (Franzen, 2018; Oakley et al., 2010; Taylor et al., 2000). These authors and others (Egan & Swedersky, 2003; Sargeant, 2003) suggest that having a personal connection to spirituality where practitioners and physical therapy students understand their own beliefs, values, and biases, may influence their comfort level and desire to carry this into their professional practice, if deemed appropriate and welcomed (Gang et al., 2022; Sargeant & Newsham, 2012).

Greater comfort in incorporating the spiritual domain into clinical practice by those with a personal spiritual connection is also supported by the central findings of this study. Consistently in all topic areas, those who considered themselves more religious/spiritual were significantly more likely to feel the importance of spirituality in all of these areas compared to those who identified as humanist/atheist/agnostic/other. This is consistent with the findings among physical therapists of the Oakley et al., (2010).

A small minority (6.8%) in this study did not see the relevance of including spirituality in patient care. Based on this study’s findings, spiritual/religious self-identity appears to have influenced the perspectives of PT practitioners in terms of the importance of including spirituality in patient care across all five spirituality topics measured. As shown earlier, almost all (95.3%) of those identifying as religious/spiritual thought it useful to consider patient spirituality compared to only 72.2% of those identifying as humanist/atheist/agnostic/other.

### Aim 2—Prior Exposure to Professional Education in Spirituality

Prior exposure to spirituality through some form of professional education appeared to have a positive impact on some of respondents’ perceptions of the role of spirituality in clinical PT practice. Although the measure of prior exposure used in this study was general, there was a significant relationship for half of the topics. A more refined measure perhaps might have provided a better assessment. Nonetheless, a positive relationship is consistent with prior research which has shown that clinicians who have had training in spiritual care are more likely to be successful in incorporating spirituality in their practice and in meeting patients’ spiritual needs (Hu et al., 2019; Paal et al., 2015). Although there is yet no direct evidence as to whether educating PT students in incorporating spirituality in patient care would impact the quality of their clinical care, participants in this study (all practicing physical therapists) and physical therapists and students in prior studies (Sargeant, 2003; Sargeant & Newsham, 2012; Tapley et al., 2012) believe that it should be included.

### Aim 3—Perceived Usefulness of Including Spirituality in Patient Care

This study found that respondents who felt that including spirituality in patient care was useful were more likely to have positive attitudes regarding including spirituality in PT practice. These findings confirm those of prior more limited studies that practicing physical therapists see the importance and relevance of considering spirituality in patient care. This was expressed by several study participants through the qualitative data focusing on the contribution to patient motivation and the provision of holistic care. Similarly, researchers have found the same attitudes regarding spirituality in patient care among healthcare professionals/students in a variety of disciplines (Jones et al., 2018; Morris, 2013; Wenham et al., 2024). These positive attitudes highlight the importance and utility of including the spiritual domain into patient-centered care.

### Clinical and Educational Implications

Overall, the findings of this study support the work of previous studies addressing spirituality in PT practice. This study is also the largest study and only the second to survey *practicing* physical therapists and addresses a recommendation by Sargeant and Newsham (2012) that such a survey be conducted to extend their original work with PT students.

Although this study did not specifically seek to identify barriers to incorporating spirituality in patient care, survey respondents raised the issue in their open-ended responses. They identified lack of time during clinical encounters and lack of preparation to address these concerns clinically since they had not received training during their academic education. These findings are consistent with those of studies mentioned earlier (Morris et al., 2012; Oakley et al., 2010; Saguil & Phelps, 2012).

Lack of preparation and desire for training was reflected by several study participants through the qualitative analysis. These findings may indicate practitioners’ lack of confidence in their ability to have such discussions with patients. Similar barriers have been reported among medical students and physicians (D’Souza, 2007; McEvoy et al., 2013), occupational therapists (Collins et al., 2009; Morris et al., 2012), and nursing students (Wu et al., 2012).

Inclusion of the spiritual domain in professional education may be necessary to bridge the gap between its demonstrated importance in relation to health and illness and developing practitioner confidence in patient care. Professional education may be a mechanism to transform attitudes, increase provider comfort level, provide specific guidance on how to take a spiritual history, appropriately use assessment tools, gather knowledge, and incorporate the spiritual domain in clinical interactions.

In fact, Sargeant (2009) and Gang and colleagues (2022) have developed educational materials for PT students which identify educational goals, content, and strategies to teach spiritual concepts which they feel would prepare students to be knowledgeable and sensitive to patient diversity. These should also allow the profession to better meet societal needs by developing practitioner confidence to foster compassionate caregiving in today’s healthcare environment. This is indeed what the field of medicine has done. As of 2014, spirituality and health competencies were developed for medical students and 90% of medical schools have included spirituality in their curricula (Koenig et al., 2010; Puchalski et al., 2014a, 2014b; Sajja & Puchalski, 2018).

As our world has changed and healthcare delivery has shifted, the ICF model has become more important (Offenbacher et al., 2011; Wade & Halligan, 2017). In response, the PT profession has broadened its scope of practice where it now acknowledges this more holistic approach to patient care by addressing the contextual factors (personal and environmental) identified in the ICF model. This is consistent with the APTA’s Core Values of Professionalism (2007)—particularly compassion/caring and professional duty (Sargeant & Newsham, 2012; Tapley et al., 2012), Guide to Physical Therapist Practice (APTA, 2014), Blueprint for Teaching Cultural Competence in PT Education (APTA, 2008), and the guiding principles of APTA’s mission statement (APTA, 2018).

Preparing PT professionals to provide holistic care by integrating spirituality into curricula is a call to a culture of excellence in PT and our professional responsibility in the twenty-first century (Jensen et al., 2019). Unfortunately, CAPTE does not specifically include spirituality in its required elements—it is theoretically addressed in cultural competency content (APTA, 2008). However, studies have shown that the majority of PT programs are not addressing spirituality in their curricula (Lavinder et al., 2013; Pitts, 2008). If spirituality is as important as the literature has shown, current curricula should specifically address spirituality in their appropriate content areas and provide foundational skills towards understanding this aspect and its impact on patient care and the recovery process.

Incorporating the spiritual domain in PT education would better prepare future professionals by increasing their comfort level in responding to patients’ spiritual concerns, understanding its relevance as a coping mechanism for addressing health challenges, and enhancing their ability to provide patient-centered holistic care. This would also help to prepare entry-level practitioners with the skills that align with accreditation standards set forth by JCAHO (2019) and CARF (2012).

### Limitations

There are a few limitations to this study. First, since most survey respondents self-identified as religious/spiritual, it is possible that they were more motivated to respond to the survey because the topic was congruent with their own approach to spirituality. Non-responders might thus differ in interest and attitudes towards spirituality from survey respondents. This would potentially create selection bias. Moreover, the categories of humanist, agnostic, and atheist were not defined in the survey. Thus, each category may contain individuals with a somewhat different understanding of the category that they self-identified with.

Indeed, there is a slightly smaller proportion of those identifying as atheist or agnostic in this study (5.5%) compared to national data reported at about 9% in 2018–19 (Pew Research Center, 2019). However, it is also possible that the decision tree used by this study may have lowered the proportion identified as atheist/agnostic if some respondents chose either of these as well as spiritual. Further encouragement of these groups about the importance of their perspective in the cover letter might have helped to motivate their participation.

Second, although this is the largest such study that has been conducted to our knowledge, it still only reflects attitudes of participating PT practitioners who are members of the APTA at that specific time and in specific practice settings (i.e., in-patient). Third, the instrument used was specifically developed by the investigators for this study and thus may have limited correspondence to instruments used in prior studies. Nevertheless, this instrument covers a specific set of topics where spirituality might apply to PT practice. The instrument showed excellent internal consistency reliability and some evidence of face and content validity, properties not assessed in many prior studies. Despite these positive initial assessments, further psychometric assessment would need to be done for future use of this measure.

Fourth, the question assessing prior exposure to spirituality through professional education was assessed as a “Yes/No” response. Future assessment of exposure should include the type of course, duration and intensity which may provide a more nuanced understanding of the influence that professional education in the spiritual domain may have on practitioners’ attitudes and perceptions. Finally, multivariate analyses were not conducted to control for possible other factors that might affect the relationships identified.

### Future Research

Although results from this study and that of others mentioned here support the notion of incorporating the spiritual domain as part of educating current and future practitioners, it would be helpful to have studies where the impact of such education on patient outcomes is assessed relative to those without such course content. Important corollary studies might address, 1) whether such professional education would increase students’ and practicing professionals’ comfort level and confidence when addressing the spiritual domain with their patients; and 2) whether patients perceive a benefit from the inclusion of spirituality in their recovery.

Finally, future research should include more extensive validation of the survey instrument used here (e.g., construct validity), so that it can be incorporated into future studies in the field.

### Conclusion

This study assessed the influence of three factors (i.e., respondents’ own belief system, prior professional education in spirituality, and perceptions of spirituality’s usefulness in patient care) on practicing physical therapists’ agreement with the role that spirituality can play in PT practice. Results showed that those whose self-identified belief system was religious or spiritual, who had prior spirituality professional education exposure, and who thought it useful to include spirituality in clinical practice, had significantly more positive attitudes toward the role of spirituality in PT practice. These findings support those of previous studies in PT and other clinical professions. Given the recognized importance of incorporating the spiritual domain into PT care, the overwhelming evidence of its relationship to positive health outcomes and the recognition by practicing professionals and students they would need training to feel competent to engage in this area. It may be time for the PT profession to consider developing curricula to educate students more explicitly in the spiritual domain.

## Supplementary Information

Below is the link to the electronic supplementary material.

Supplementary file1 (DOCX 91 KB)
