# Supplementary material for: Revisiting Spirituality in Physical Therapy Practice: Perceptions of US Practitioners
Source: J Relig Health. 2025 Dec 29;65(3):2672–98. doi: 10.1007/s10943-025-02502-4 (PMC13219135; doi:10.1007/s10943-025-02502-4)
Supplement: Supplementary file 1 — Supplementary file1 (DOCX 91 KB) [file 10943_2025_2502_MOESM1_ESM.docx]

**Perception of Spirituality in PT Practice**

Appendix: Survey

*For the purpose of this study,* ***spirituality*** *is considered an internal experience that provides a sense of meaning and purpose in life and connectedness with the self and others.* ***Religion*** *is an extrinsic organized set of standards and beliefs· grounded in institutional standards.*

*Please respond to the following:*

**Section A: Demographics**

What is your:

I. Age

___20 - 24 ___25 - 34 ___35-44 ___45-54 ___55-64 ___65 +

1. Gender

___Male ___Female

1. How many years have you been working as a physical therapist in the rehabilitation setting?

___Less than 1 yr

___ 2-5 yrs

___6-10 yrs

___ 11-15 yrs

___16-20 yrs

___ More than 20 yrs

1. I consider myself: (please check all that apply).

___ Religious

___ Spiritual

___ An agnostic

___ An atheist

___ A humanist

___ Other (please specify)

| **Section B: Descriptions of Spirituality** | **Strongly Agree** | **Agree** | **Undecided** | **Disagree** | **Strongly Disagree** |
| --- | --- | --- | --- | --- | --- |
| I. Spirituality is a fundamental part of each person. | □ | □ | □ | □ | [_] |
| 2. Spirituality is an integration of body, mind and spirit. | □ | □ | □ | □ | □ |
| 3. Spirituality is concerned with a personal interpretation of life and the inner strength of  people. | □ | □ | □ | □ | □ |
| 4. Spirituality is considered a life long process. | □ | □ | □ | □ | □ |
| 5. Spirituality is a component in quality of life measures. | □ | □ | □ | □ | □ |

| **Section C: Spirituality and PT Practice** | **Strongly**  **Agree** | **Agree** | **Undecided** | **Disagree** | **Strongly**  **Disagree** |
| --- | --- | --- | --- | --- | --- |
| I. Knowledge of spirituality will improve my  understanding of patient-centered care. | □ | □ | □ | □ | □ |
| 2. Considering spirituality enhances my treatment  approach by caring for the whole person: body, mind and spirit. | □ | □ | □ | □ | □ |
| 3. Including spirituality negatively affects my productivity. | □ | □ | □ | □ | □ |
| 4. Knowledge of spirituality develops my sensitivity  toward the patient as a person. | □ | □ | □ | □ | □ |
| 5. Addressing spirituality as a part of total patient  care will help me to set realistic patient-centered goals. | □ | □ | □ | □ | □ |
| 6. Including spirituality in physical therapy is  beyond the scope of practice. | □ | □ | □ | □ | □ |
| 7. Spirituality can be a motivator in the  rehabilitation process. | □ | □ | □ | □ | □' l |
| 8. Spiritual needs should be addressed in the  rehabilitation process. | □ | □ | □ | - □ | □ |
| 1. Spirituality care includes:    - Active listening | □ | □ | □ | □ | □ |
| - Talking | □ | □ | □ | □ | □ |
| - Being present | □ | □ | □ | □ | □ |
| - Accepting patient's spiritual concerns | □ | □ | □ | □ | □ |
| - Exploring patient's spiritual concerns | □ | □ | □ | □ | □ |
| 10. I need to recognize my patient's spiritual needs and refer them to appropriate disciplines when their needs arc beyond my scope of practice. | □ | □ | □ | □ | □ |

| **Section D: Spirituality and the Patient** | **Strongly Agree** | **Agree** | **Undecided** | **Disagree** | **Strongly Disagree** |
| --- | --- | --- | --- | --- | --- |
| I. Spirituality allows healing even when complete recovery is not possible. | □ | □ | □ | □ | □ |
| 2. Awareness of patient's spiritual dimension opens the door for constructive dialog for their  condition. | □ | □ | □ | □ | □ |
| 3. Incorporating spirituality in patient care improves function and the overall recovery process. | □ | □ | □ | □ | □ |
| 1. Recognizing the patient's spiritual needs can facilitates the recovery process by developing:    - Psychological adjustments | □ | □ | □ | □ | □ |
| - Inner strength | □ | □ | □ | □ | □ |
| - Emotional support | □ | □ | □ | □ | □ |
| 5. A close link exists between spirituality and perception of disability. | □ | □ | □ | □ | □ |

**Section E: Spirituality and the Therapist the Physical**

**Strongly Agree**

□

□

□

□

□

□

□

**Agree**

**Undecided Disagree**

I

1. I am aware of my spirituality.
2. As a clinician I take time to get to know the person in the patient.
3. In my clinical practice, I allow patients to find meaning of their circumstances without questions.
4. Spiritual care of my patient's improves my personal and professional growth.
5. Integrating spirituality in clinical practice is based on quality of care and ethical principles.
6. Spirituality is a significant and important domain of patient's adjustment.
7. , I routinely ask questions about patient's spirituality during the therapeutic process.

□

□

□

□

□

□

□

□

□

□

□

□

□

□

-

□

□

□

□

□

□

□

**Strongly Disagree**

□

□

□

□

□

□

□

| **Section D: Continued** | **Strongly Agree** | **Agree** | **Undecided** | **Disagree** | **Strongly Disagree** |
| --- | --- | --- | --- | --- | --- |
| 6. Incorporating spirituality in the plan of care gives  patient's meaning and purpose in their day, life and relationships. | [.J | D | □ | □ | lJ |
| 7. Spirituality is one way to help my patients put their past in perspective. | LJ | [] | IJ | □ | [] |
| 1. Discussing patient's spiritual needs facilitates:    - Independence | [ l | [l | □ | □ | □ |
| - Control | r ·1  .J | 11 | [l | n | [7 |
| - Comfort | J | [ l | [] | □ | [] |
| - Coping with pain | *[* I | Ll | □ | LJ | f"l |
| 9. Understanding spirituality in clinical practice promotes trusting relationships between me and my patient's. | I i  L' | 1· l | ' ,1 | n | r J |
| 10. Spirituality helps patients to view disease or illness as a meaningful challenge in their own development. | r , | u | Ll | D | Ir._·1 |
| 1. Patients' spiritual needs include:    - Trust | l l | □ | □ | □ | LJ |
| - Awareness of self | LJ | [j | □ | □ | □ |
| - Sense in purpose in life | [I | □ | [J | □ | □ |
| - Awareness of mortality | □ | □ | □ | □ | □ |
| - Vulnerability | □ | □ | □ | D | □ |

*If* ***spirituality*** *is integrated in your clinical practice, please respond to the next section. If not,*

*please skip to Section G:*

| **Section F: Spirituali y and Outcomes**  ·' | **Strongly Agree** | **Agree** | **Undecided** | **Disagree** | **Strongly Disagree** |
| --- | --- | --- | --- | --- | --- |
| I. Spirituality is a powerful resource for  adjustment in patients with disability. | □ | □ | □  I I | □ | □'  I |
| 2. Spirituality affects a patient's attitude. | □ | □ | □ | □ | □ |
| 1. Spirituality impacts:    - The patient's length of stay | □ | □ | □ | □ | □ |
| - The patient's ability to cope with pain | □ | □ | □ | □ | □ |
| 1. Spirituality has the ability to:    - Alter the course of illness/disease process   -- - ---. ·---·-····--------·- -·------·--- ------------------ | □ | □ | □ | □ | □ |
|  | □ | □ | □ | □ | □ |
| - Change the perception of self and the world |  |  |  |  |  |
| - Change the use of medications | □ | □ | □ | □ | □ |
| 1. Spirituality facilitates:    - Function | □ | □ | □ | □ | □ |
| - Compliance | □ | □ | □ | □ | □ |
| - Independence | □ | □ | □ | □ | □ |
| - The relationship between therapist and patient | □ | □ | □ | □ | □ |

I

**Section G: Spiritual Background**

1. Information on spirituality was included in my physical therapy education.

___Yes ___No ___Unsure

1. I have attended continuing education regarding spirituality.

___Yes ___No

1. Do you think it is useful for the physical therapist to consider the spiritual dimension of the patient? ___Yes ___No Please explain:
2. What does spirituality mean to you?

Please add any additional comments you might have on this topic:

**THANK YOU VERY MUCH FOR YOUR PARTICIPATION**
